# Transcriptome analysis of the hepatopancreas from the *Litopenaeus vannamei* infected with different flagellum types of *Vibrio alginolyticus* strains

**DOI:** 10.3389/fcimb.2023.1265917

**Published:** 2023-11-21

**Authors:** Jingwen Zhang, Kaifang Liu, Xiaoxiao Gong, Na Zhang, Yanhua Zeng, Wei Ren, Aiyou Huang, Hao Long, Zhenyu Xie

**Affiliations:** ^1^Hainan Provincial Key Laboratory for Tropical Hydrobiology and Biotechnology, Hainan University, Haikou, China; ^2^Laboratory of Development and Utilization of Marine Microbial Resource, Hainan University, Haikou, China; ^3^School of Fisheries, Xinyang Agriculture and Forestry University, Xinyang, China; ^4^Collaborative Innovation Center of Marine Science and Technology, Hainan University, Haikou, China

**Keywords:** *Vibrio alginolyticus*, flagella, *flhG*, *Litopenaeus vannamei*, transcriptome

## Abstract

*Vibrio alginolyticus*, one of the prevalently harmful Vibrio species found in the ocean, causes significant economic damage in the shrimp farming industry. Its flagellum serves as a crucial virulence factor in the invasion of host organisms. However, the processes of bacteria flagella recognition and activation of the downstream immune system in shrimp remain unclear. To enhance comprehension of this, a Δ*flhG* strain was created by in-frame deletion of the *flhG* gene in *V. alginolyticus* strain HN08155. Then we utilized the transcriptome analysis to examine the different immune responses in *Litopenaeus vannamei* hepatopancreas after being infected with the wild type and the mutant strains. The results showed that the Δ*flhG* strain, unlike the wild type, lost its ability to regulate flagella numbers negatively and displayed multiple flagella. When infected with the hyperflagella-type strain, the RNA-seq revealed the upregulation of several immune-related genes in the shrimp hepatopancreas. Notably, two C-type lectins (CTLs), namely galactose-specific lectin nattectin and macrophage mannose receptor 1, and the TNF receptor-associated factor (TRAF) 6 gene were upregulated significantly. These findings suggested that C-type lectins were potentially involved in flagella recognition in shrimp and the immune system was activated through the TRAF6 pathway after flagella detection by CTLs.

## 1 Introduction

Pacific white shrimp (*Litopenaeus vannamei*) is highly favored in China and other countries for its abundant protein and low-fat content, making it the most popular shrimp species for consumption (Li et al., 2021). As the breeding industry increased rapidly, epidemic diseases have become one of the major risk factors for shrimp aquaculture in recent years (Yu et al., 2022). *Vibrio alginolyticus* and other marine-dwelling *Vibrio* species are conditional pathogens that cause white feces syndrome (WFS), acute hepatopancreatic necrosis disease (AHPND), and other vibrioses in shrimps (Janda et al., 2015; Baker-Austin et al., 2018; Ngo et al., 2020; Foysal et al., 2021; Kumar et al., 2021; Shen et al., 2021; Munkongwongsiri et al., 2022).

When pathogenic *Vibrio* species or other pathogens infect the host, the immune system will be activated by recognizing several molecules called pathogen-associated molecular patterns (PAMPs). These molecules are crucial for the pathogens’ virulence and survival (Kumar et al., 2011). The host’s pattern recognition receptors (PRRs), including Toll-like receptors (TLRs), RIG-I-like receptors (RLRs), NOD-like receptors (NLRs), and DNA receptors, sense these PAMPs (Medzhitov, 2007; Kumar et al., 2009). After the PAMPs recognized by PRRs, the immune system responds quickly to eliminate the pathogens through several signaling pathways (Li et al., 2009; Fitzgerald and Kagan, 2020).

Being a major PAMP, the bacterial flagellum is also an important virulence factor for its function in motility, chemotaxis, adhesion, biofilm formation, and secretion (Duan et al., 2013; Chaban et al., 2015; Rossez et al., 2015). In mammals and teleost fish, the bacterial flagella recognition and signaling are through TLR5, NLRC4, and NAIP5, and that activation of these receptors mobilizes nuclear factor NF-kappaB and stimulates the production of tumor necrosis factor-alpha (TNF-α) (Feuillet et al., 2006; Zhao and Shao, 2015; Fitzgerald and Kagan, 2020). Some pathogens also develop strategies to avoid the recognition of the flagella and escape the host immune system clearance. During infection, *S. typhimurium* significantly reduces the expression of genes involved in flagellar machinery and chemotaxis when present inside macrophages (Eriksson et al., 2003). By day 3 after feeding, the motility of *E. coli* introduced into the mouse gut decreased by 45-50%, and by day 15, it dropped further to 80-90% loss of motility (Gauger et al., 2007). Other methods for evading detection by the host immune system include regularly alternating the expression of flagellin proteins (phase variation), enabling different subsets of the population to express flagella (bistability). Additionally, modifying the structure of flagellin proteins to make them unrecognizable to TLR5 (flagellin modification) and adding post-translational modifications to flagellins to mask target sites (glycosylation) are also effective strategies (Chaban et al., 2015). Yet, there is scant research about how crustacean, including shrimps, recognizes bacteria flagella and actives their immune system. Several TLRs have been found in shrimps and carbs, but none has been confirmed to respond to flagella (Habib and Zhang, 2020).

In this research, we injected the wild-type and mutant strains of *V. alginolyticus* strain HN08115 with different flagella phenotypes into *L. vannamei* and analyzed the different gene expression patterns in shrimp hepatopancreas. Our results might help explain how shrimps’ immune systems recognize bacterial flagella and activate the immune system through signaling pathways.

## 2 Materials and methods

### 2.1 Bacterial strains and plasmids

The strains and plasmids are listed in **Table 1**. The *V. alginolyticus* strain HN08155 was cultured in 2216E medium at 30°C. *E. coli* strain β2163 was cultured in LB broth with 0.3 mM DAP at 37°C. The medium was added with chloramphenicol (50μg/ml), or ampicillin(100μg/ml) according to the plasmid feature.

**Table 1:** Bacterial strains and plasmids used in this study.

Plasmids and strains	characteristics	References
Plasmids		
pDM4	Cm^R^, *sacBR*, Suicide plasmids containing replicons of π -dependent protein oriP6K	Laboratory collection
pDM4-*flhG*	Cm^R^, PDM4 with *flhG* gene fragment missing was inserted into the frame	This study
E. coli		
DH5α	The recipient of DNA manipulation	Laboratory collection
β2163	(F-)RP4-2-Tc::Mu△dapA☹erm-pir), Bacterial conjugation donor strain	Laboratory collection
β2163-pDM4-*flhG*	Cm^R^, Introduction of pDM4-*flhG* plasmid into β2163	This study
V. alginolyticus		
HN08155	Wild type stain	Isolated from the previous study
△*flhG*	*flhG* gene knocked out the strain of HN08155	This study

### 2.2 Construction of flhG mutant

About 600 bp of the *flhG* gene’s upstream and downstream regions were amplified by PCR separately. Then the two fragments were joined together by overlap extension PCR. Primers used in amplifications are listed in **Table 2**. The junction fragment was inserted into the suicide vector pDM4 at the *XbaI* site to constructed recombinant plasmid pDM4-*flhG*. The plasmid pDM4-*flhG* was transformed into *E. coli* β2163 and introduced into *V. alginolyticus* HN08155 by conjugation. LBS agar containing 10% sucrose was used to screen the double-crossover recombinant. The △*flhG* mutate strain was confirmed by PCR and sequencing.

**Table 2:** Primers.

Primers name	Primers sequence (5’-3’)
*flhG*-T-F	GCTCAGAGCAATGCTGATTA
*flhG*-T-R	ATCGTCCGCTTCTTGAGTGT
*flhG*-UF	GAAGATCTAAACAACCACCGTTGCGAAG
*flhG-*UR	TCTGCGAATTCGGTACGATTGCTTGCTTGATCGTGTATCA
*flhG-*DF	TGATACACGATCAAGCAAGCAATCGTACCGAATTCGCAGA
*flhG-*DR	CCCTCGAGGATTCGCTTACGCCTAACAC
pDM4-TF	CACAGGAACACTTAACGGCT
pDM4-TR	TCCTGTTCAGCTACTGACGG
pBAD-R-F	CCATATGGGAATTCGAAGCT
pBAD-R-R	TCGAGCTCGGATCCATGGTTA
pBAD-*flhG*-F	TAACCATGGATCCGAGCTCGAATGACTGAGAATATGATACA
pBAD-*flhG*-R	AGCTTCGAATTCCCATATGGTTCACCAAAAGGGTCCTCTG
pBAD-T-F	CGTCACACTTTGCTATGCCA
pBAD-T-R	AATCTTCTCTCATCCGCCAA

### 2.3 Flagellum observation by transmission electron microscope

The *V. alginolyticus* HN08155 wild-type (WT) and △*flhG* strains were cultured until the OD600 values were approximately 1.0. The cells were washed and gently resuspended with normal saline solution (0.9% NaCl). 10μl of the resuspension was added to a 300-mesh carbon-coated Formvar grid (Electron Microscopy Science, Hatfield, Pennsylvania) and negatively stained with a 2% (w/v) phosphotungstic acid solution. The JEOL JEM-2100 transmission microscope was used to acquire the images.

### 2.4 Shrimp rearing and sample collection

Healthy *L. vannamei* (12 ± 2g) was purchased from a shrimp farm in Dongfang, Hainan, China. The shrimp were maintained in tanks with aerated seawater (30 ppt, 26 ± 2°C) for one month. The shrimps were randomly separated into two groups and were injected with the *V. alginolyticus* HN08155 WT and △*flhG* strains into the tail muscle to a final concentration of 8 x 10^4^CFU/g. After 4 hours of the injection, the hepatopancreases of three random shrimps of each group were collected as one sample and frozen in liquid nitrogen for transcriptome analysis. Three replicate samples were collected from each group.

### 2.5 cDNA library construction and sequencing

Total RNA from the hepatopancreas sample was extracted from the tissue using TRIzol® Reagent according to the manufacturer’s instructions (Invitrogen) and genomic DNA was removed using DNase I (Takara). RNA degradation and contamination were monitored on 1% agarose gels. Then RNA quality was determined by 2100 Bioanalyser (Agilent Technologies) and quantified using the ND-2000 (NanoDrop Technologies). Only high-quality RNA sample (OD260/280 = 1.8~2.2, OD260/230≥2.0, RIN≥8.0, 28S:18S≥1.0, >1μg) was used to construct the sequencing library.

RNA purification, reverse transcription, library construction, and sequencing were performed at Shanghai Majorbio Bio-pharm Biotechnology Co., Ltd. (Shanghai, China) according to the manufacturer’s instructions (Illumina, San Diego, CA). The transcriptome library was prepared following the TruSeq TM RNA sample preparation Kit from Illumina (San Diego, CA) using 1μg of total RNA. Shortly, messenger RNA was isolated according to the polyA selection method by oligo(dT) beads and then fragmented by fragmentation buffer first. Secondly, double-stranded cDNA was synthesized using a SuperScript double-stranded cDNA synthesis kit (Invitrogen, CA) with random hexamer primers (Illumina). Then the synthesized cDNA was subjected to end-repair, phosphorylation, and ‘A’ base addition according to Illumina’s library construction protocol. Libraries were size selected for cDNA target fragments of 300 bp on 2% Low Range Ultra Agarose followed by PCR amplified using Phusion DNA polymerase (NEB) for 15 PCR cycles. After being quantified by TBS380, the paired-end RNA-seq sequencing library was sequenced with the Illumina NovaSeq 6000 sequencer (2 × 150 bp read length).

### 2.6 Quality control and read mapping

The raw paired-end reads were trimmed and quality controlled by fastp (https://github.com/OpenGene/fastp) with default parameters. Then clean reads were separately aligned to the reference genome (GCF_003789085.1, https://www.ncbi.nlm.nih.gov/assembly/GCF_003789085.1/) with orientation mode using HISAT2 (http://ccb.jhu.edu/software/hisat2/index.shtml) software (Zhang et al., 2019). The mapped reads of each sample were assembled by StringTie (https://ccb.jhu.edu/software/stringtie/) in a reference-based approach.

### 2.7 Differential expression analysis and functional enrichment

To identify DEGs (differential expression genes) between two different samples/groups, the expression level of each gene was calculated according to the transcripts per million reads (TPM) method. RSEM (http://deweylab.biostat.wisc.edu/rsem/) was used to quantify gene abundances. Essentially, differential expression analysis was performed using the DESeq2, DEGs with |log2(foldchange)| ≥ 1 and P-adjust ≤ 0.05 (DESeq2/edgeR/Limma)/P-adjust ≤ 0.001 (DEGseq)/Prob > 0.8 (NOIseq) were considered to be significantly differentially expressed genes. In addition, functional-enrichment analyses including GO (Gene Ontology, http://www.geneontology.org) and KEGG (Kyoto Encyclopedia of Genes and Genomes, http://www.genome.jp/5eg/) were performed to identify which DEGs were significantly enriched in GO terms and metabolic pathways at P-adjust ≤ 0.05 compared with the whole-transcriptome background. GO functional enrichment and KEGG pathway analysis were carried out by Goatools (https://github.com/tanghaibao/Goatools) and KOBAS (http://kobas.cbi.pku.edu.cn/home.do).

### 2.8 DEG validation using quantitative real-time PCR

To validate the RNA-Seq data, qRT-PCR was carried out on a LightCycler 96 real-time PCR system (Roche Molecular Systems, Inc) using SYBR Green Mix (Vazyme, China). The information of primers was shown in **Table 3**, and β-actin was selected as a reference gene. The 2^−ΔΔCt^ method was used to analyze the target gene’s relative expression (fold changes). The amplification efficiency(E) was calculated on standard curves using 10-fold dilutions of cDNA, E=10^-1/slope^-1. All samples were run in triplicate.

**Table 3:** Primers used for the qRT-PCR.

Primers name	Primers sequence (5’-3’)	Functions	Product fragment length	Amplification efficiency (E)
β-actin-F	GCCCATCTACGAGGGATA	Reference gene	160bp	98.8%
β-actin-R	GGTGGTCGTGAAGGTGTAA			
LOC113824731-F	CCTCATGTTGACGAGAGAAGCCT	crustacyanin-C1 subunit-like	179bp	96.1%
LOC113824731-R	GCGTCAATACCAGTGGATCTGACG			
LOC113828299-F	TGCTGTAGTGCTGTGTTTCGCC	cyclin-dependent kinase inhibitor 1C-like	166bp	97.6%
LOC113828299-R	GGCGTTGAGGGCGTTGATCTG			
LOC113810339-F	ACAGGTGGTGGACCAGTTCAAC	TNF receptor-associated factor 6-like	187bp	105.4%
LOC113810339-R	CGTCCAGGTCGTCGAAGAAGTC			
LOC113807693-F	TTCAGGTACGCATGAAGCCTCCT	cytochrome P450 2L1-like	193bp	103.5%
LOC113807693-R	CAAGCCATACACCCAGGACAGTC			
LOC113822335-F	CCGACCATCCAGGATTACTTCGG	nitrate reductase [NADH] 1-like	187bp	98.2%
LOC113822335-R	CGGAGATGAATTTGGCTTGCCAG			
LOC113823154-F	GGAATCGAAGGCGTTGAGATGCC	cytochrome P450 4c3-like	185bp	97.2%
LOC113823154-R	CTGTCCCAAACTGCGTCGAATT			

## 3 Results

### 3.1 △flhG mutant construction and flagella observation

**Figure 1:**
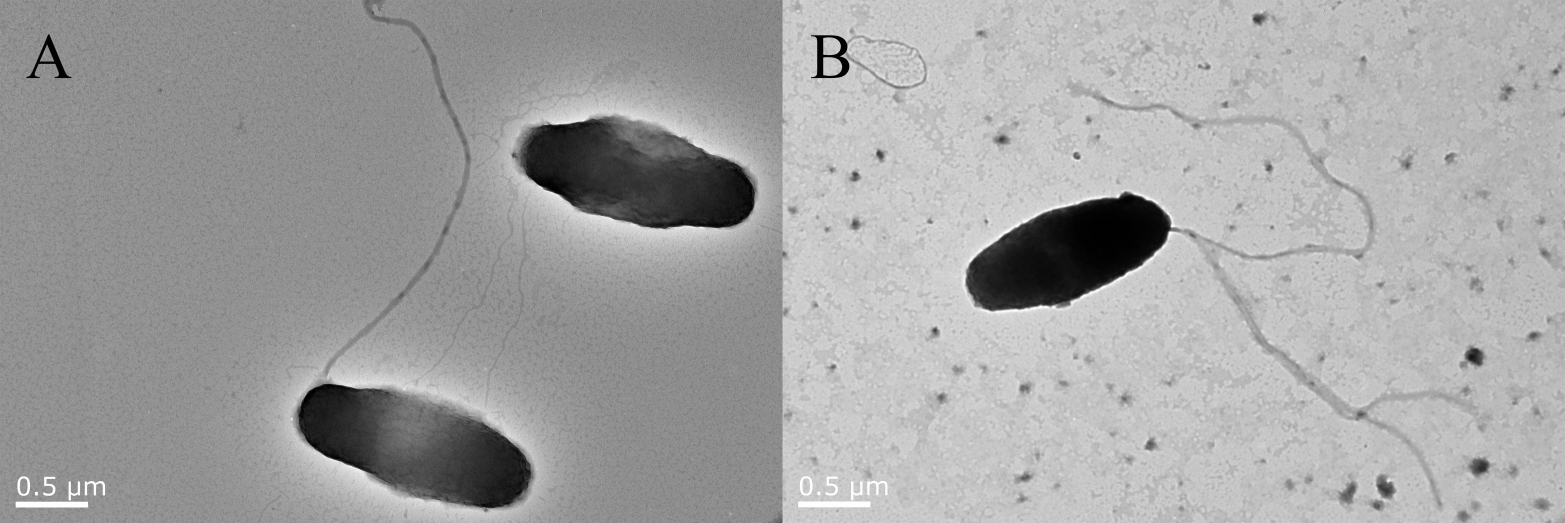
Flagella structure of WT and △*flhG* strains. *V. alginolyticus* strain HN08155 flagella structure under TEM scan. **(A)** WT strain with a single flagellum. **(B)** △*flhG* strain with multiple flagella.

The *flhG* gene of *V. alginolyticus* strain HN08155 has been knocked out successfully by in-frame deletion. The △*flhG* strain has been verified by PCR assay (data not shown). The external structure of *V. alginolyticus* strain HN08155 WT and △*flhG* strains was observed using a transmission electron microscope (TEM) to analyze the impact of the *flhG* gene on flagella formation. The WT strains have a single flagellum on their poles (**Figure 1A**), while the *flhG* mutant strains have several polar flagella to form a hyper flagella structure (**Figure 1B**).

### 3.2 Sequencing and *de novo* assembly

Six cDNA libraries of hepatopancreas from the HN08155 WT strain infected group and △*flhG* strain infected group were constructed to perform transcriptome analysis of *L. vannamei* in response to different *V. alginolyticus* strains. A total of 307,384,620 clean reads were obtained, with an average mapping rate of 88.08~89.59% to the reference genome and a Q20% higher than 98.4%. The average error rate was 0.024% (**Table 4**). These results indicate that the sequence data have good quality for further analysis. RNA-Seq data were deposited to the US National Center for Biotechnology Information (NCBI) (accession number PRJNA1030993).

**Table 4:** Summary of the sequencing data.

Sample	Clean reads	Total mapped	Error rate (%)	Q20 (%)	GC content (%)
WT1	55857574	49860332 (89.26%)	0.0236	98.58	53.62
WT2	53524830	47951382 (89.59%)	0.0238	98.49	53.63
WT3	52437752	46575267 (88.82%)	0.0237	98.56	54.38
△*flhG*1	48595002	42802842 (88.08%)	0.0236	98.56	53.56
△*flhG*2	47529596	42301498 (89.0%)	0.0254	97.92	53.57
△*flhG*3	49439866	43797446 (88.59%)	0.0242	98.3	50.88

Q20: Percentage of the bases with Qphred > 20 (error rate < 1%).

### 3.3 Functional annotation and classification of unigenes

All unigenes were searched against the GO, KEGG, COG, Nr, Swissprot, and Pfam databases. In this study, 17,291 unigenes were annotated. Among them, a total of 17,291, 9,075, 5,808, 9,184, 13,943, 8,022, and 9,190 unigenes were annotated in the GO, KEGG, COG, Nr, Swissprot, and Pfam database, respectively (**Table 5**).

**Table 5:** Summary of function annotation.

Database	Number of annotated unigenes	Ratio (%) of annotated unigenes
All	17,291	100.00
GO	9,075	52.48
KEGG	5,808	33.59
COG	9,184	53.11
NR	13,943	80.64
Swiss-Prot	8,022	46.40
Pfam	9,190	53.15

### 3.4 The analysis and functional annotation of the DEGs

**Figure 2:**
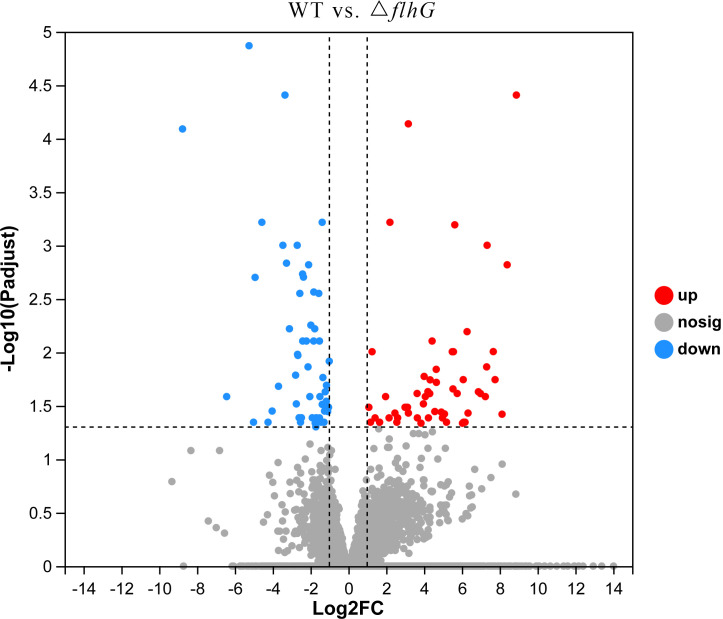
Volcano diagram. Volcano diagram of differentially expressed genes in *L. vannamei* hepatopancreas, △*flhG* group vs. WT group. The x-axis indicates the fold change, and the y-axis indicates the statistical significance of the differences.

**Figure 3:**
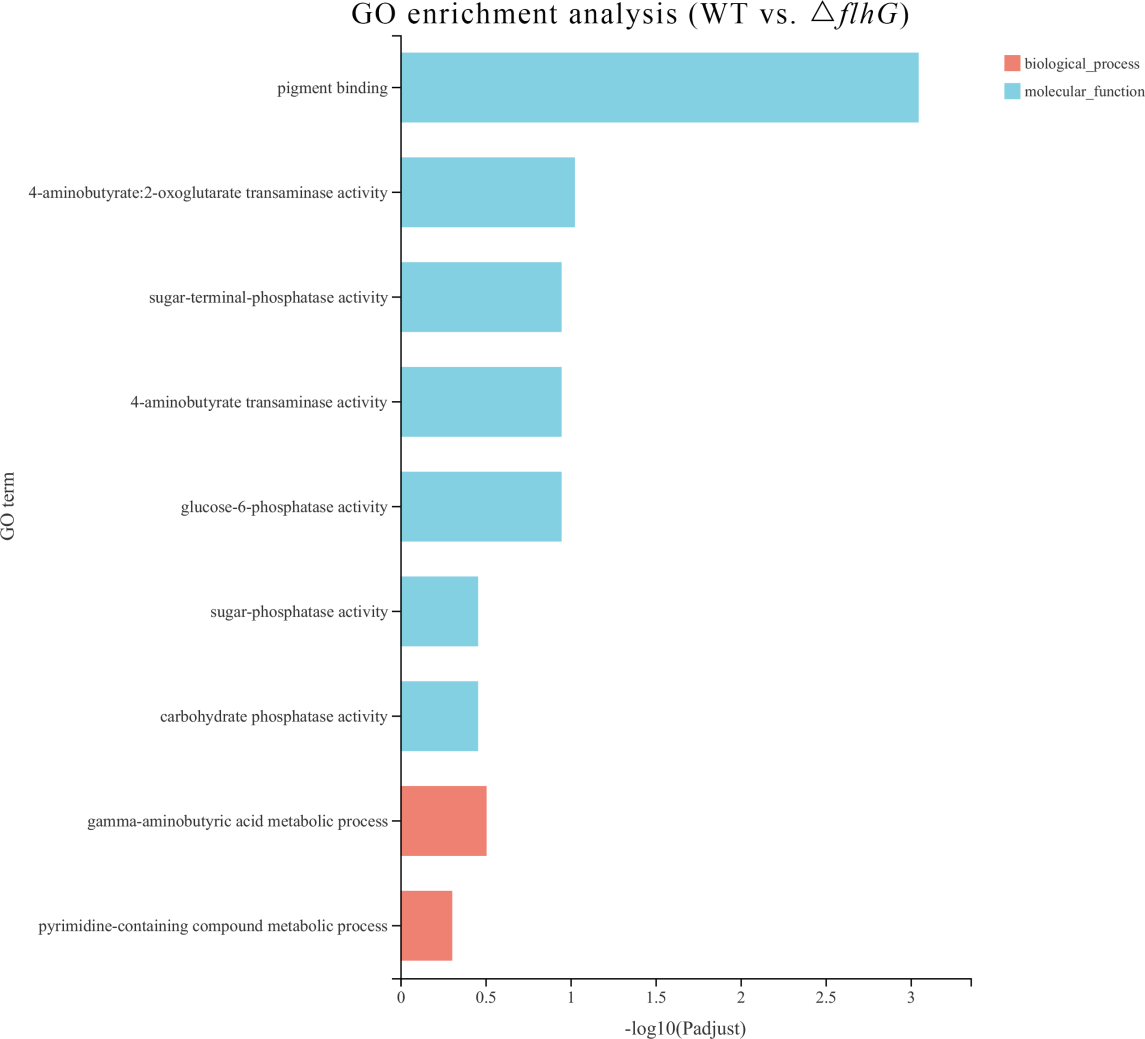
Enriched Gene ontology (GO) terms for DEGs in WT vs. △*flhG*. The x-axis indicates the significance level of enrichment, and the y-axis indicates the GO term.

**Figure 4:**
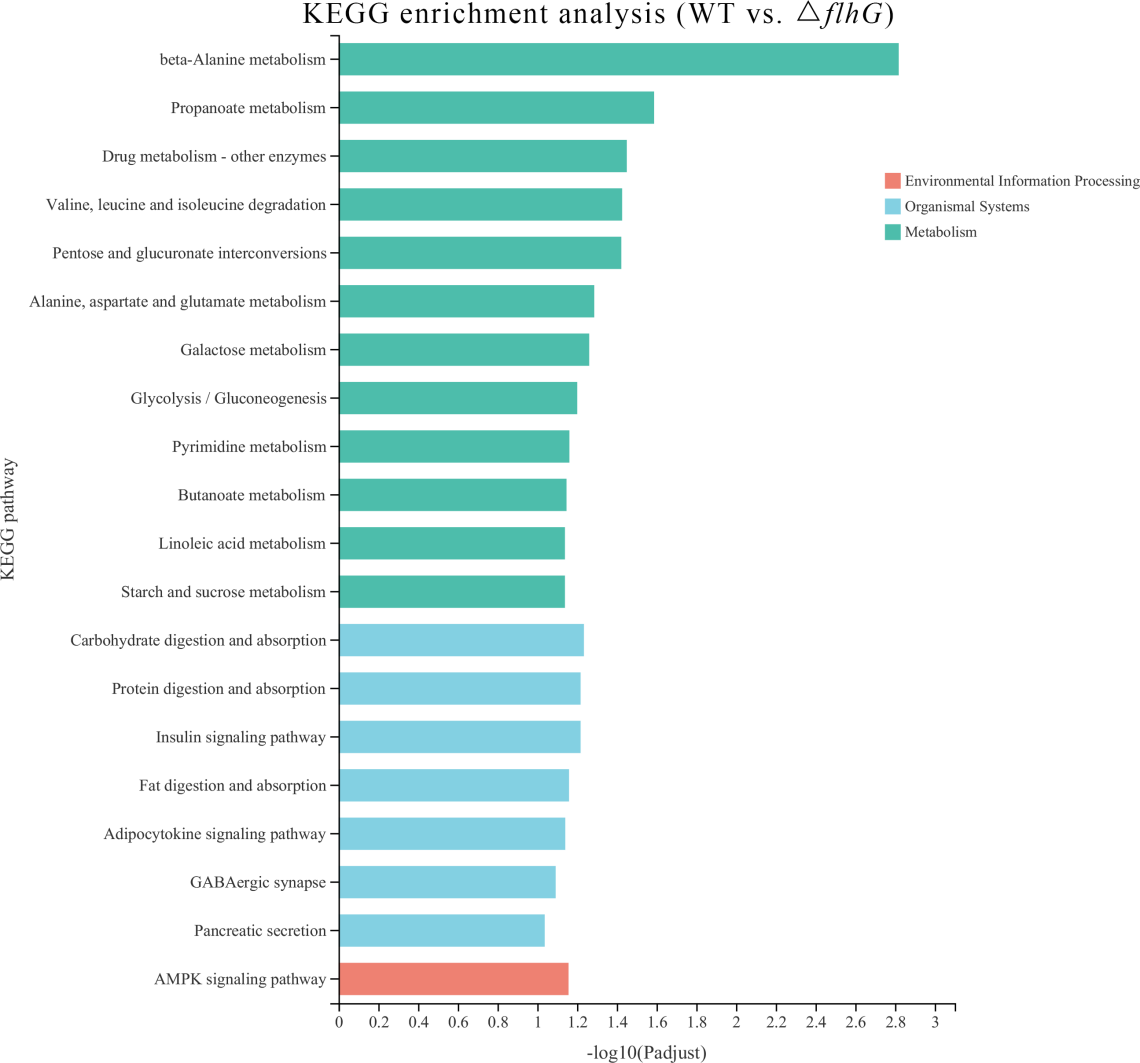
KEGG pathways enriched by the DEGs. The x-axis indicates the significant level of enrichment, and the y-axis indicates the KEGG pathway.

The differentially expressed genes of hepatopancreas response to *flhG* mutant strains (△*flhG* group) were compared to hepatopancreas response to WT mutant strains (WT group). In total, 104 significant DEGs were found in △*flhG* vs. WT, containing 52 up-regulated genes and 52 down-regulated genes (**Figure 2**, **Supplementary Table 1**). The DEGs were annotated using GO and KEGG databases. The GO enrichment revealed that DEGs between shrimps infected with different strains were significantly enriched in Molecular Function (MF) and Biological Process (BP), especially in the pigment binding term (GO:0031409) (**Figure 3**). The KEGG enrichment show that DEGs were assigned to 3 special KEGG pathways, including metabolism, organismal systems, and environmental information processing. In metabolism, beta-alanine metabolism, propanoate metabolism, and drug metabolism were the top pathways that enriched most DEGs. In organismal systems, carbohydrate digestion and absorption, protein digestion and absorption, and insulin signaling pathway were the top pathways that enriched most DEGs. In environmental information processing, the DEGs are enriched in the AMPK signaling pathway (**Figure 4**).

### 3.5 Transcriptome results validation by qRT-PCR

**Figure 5:**
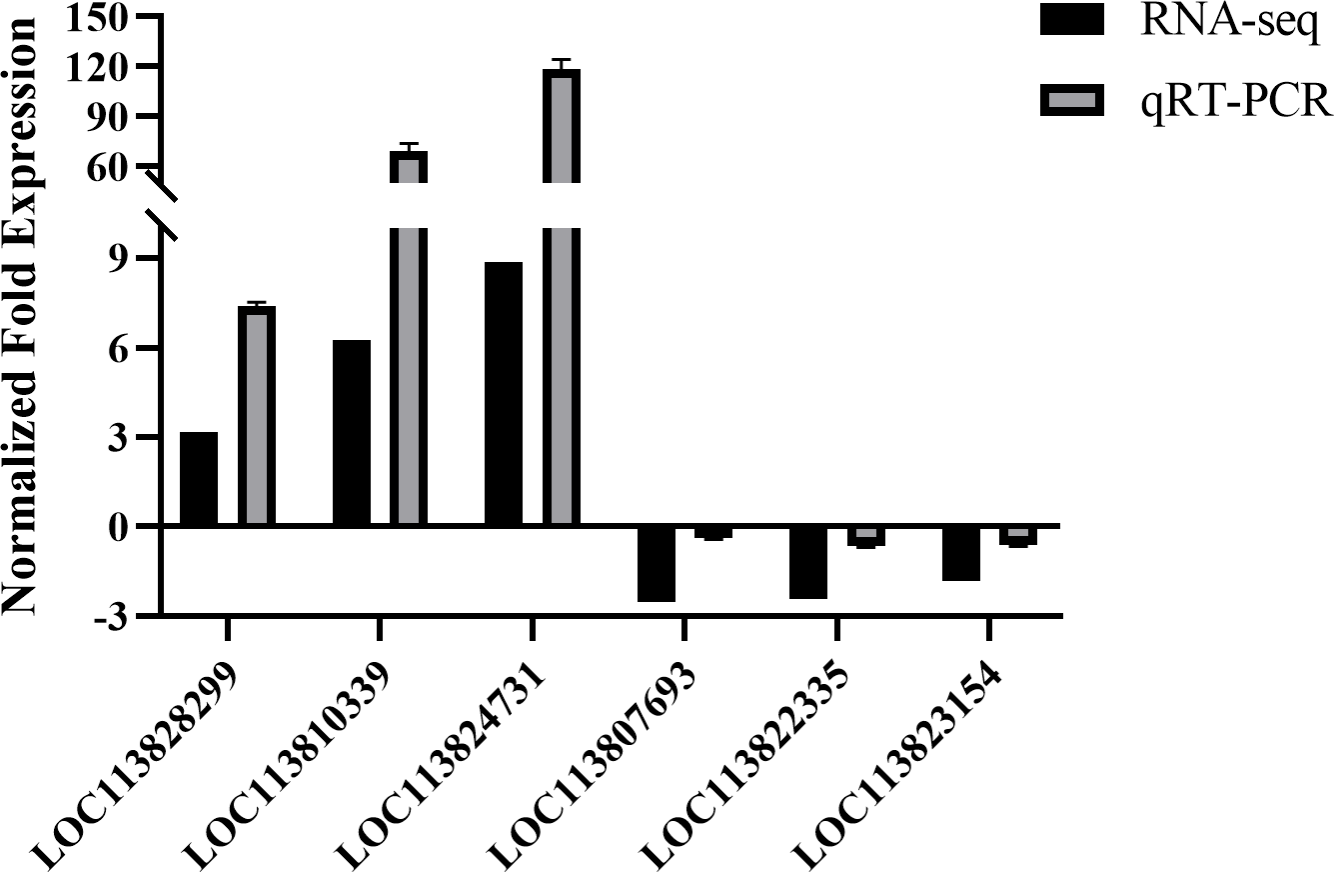
qRT-PCR validation. Comparison of the expression profiles of selected genes as determined by RNA-seq and qRT-PCR.

qRT-PCR were used to verify the DEGs gene expression. The relative expressions of several DEG genes are shown in **Figure 5**. The expression levels of selected genes were consistent with the RNA-seq data, suggesting that the transcriptome analysis results are reliable.

## 4 Discussion

Although there is only one polar flagellum on their rod-shaped cells, *Vibrio* species are commonly mobile in water, and flagellar motility is important for them to survive and infect the host (Zhu et al., 2017; Khan et al., 2020). *V. alginolyticus* is one of the pathogenic *Vibrio* spp. commonly found in diseased marine cultured species. In *V. alginolyticus* the assembly of the polar flagellum is promoted by FlhF and inhibited by FlhG which is a MinD homolog and an ATPase (Ono et al., 2015; Takekawa et al., 2016). *FlhG* gene-deficient bacteria will lose the negative regulation ability of flagella and result in the hyperflagellated form (Gulbronson et al., 2016). Since flagella is a major PAMP molecules recognized by the immune system, down-regulation of the flagella is one of the strategies used by pathogens to survive inside the host (Chaban et al., 2015). The role of *V. alginolyticus flhG* in host invasion remains unclear to us. In this research, we deleted the *flhG* gene in a *V. alginolyticus* strain HN08155 which was isolated in our previous research from diseased groupers (Xie et al., 2020). The *flhG* mutant strains of HN08155 exhibit hyper flagella according to our TEM results (**Figure 1B**), suggesting that the mutants have lost their ability to negatively regulate the number of flagella and might not escape the immune recognition by inhabiting the flagella.

The transcriptome technology is an efficient method to study the pathways or gene expression patterns in shrimps and other invertebrates. Peng et al. studied the molecular mechanism of *L. vannamei*’s cold tolerance by hepatopancreas transcriptomic analysis (Zhuo et al., 2021). Zhang et al. compared the different gene expressions before and after *L. vannamei* was infected by decapod iridescent virus 1 (DIV1) and found that TPI-like genes were crucial in DIV1 infection (Liao et al., 2020). Lin et al. also discovered several unique immune-related genes from *V. parahaemolyticus* infected *L. vannamei* by RNA-seq (Qin et al., 2018). Although numerous reports have focused on the gene expression changes of shrimps after being challenged with *Vibrio* spp., the signaling pathways in which the shrimp immune system recognizes the bacteria flagellum and starts the activation are still unknown to us. In this study, we have injected *V. alginolyticus* strains with different flagella regulation abilities into *L. vannamei* and explored the different gene expression responses of the shrimp hepatopancreas by transcriptome. The wild-type strains of *V. alginolyticus* HN08155 have a full flagella regulation function and may possess one or zero polar flagellum after being infected into the host tissue. On the contrary, the *flhG* mutant strains will have multiple flagella under all conditions. In this case, the different gene expression profiles of the shrimp response to the two types of strains might help us to understand the molecule mechanisms of flagella recognition and signaling transduction in *L. vannamei.* The transcriptome analysis identified a total of 104 DEGs, with 52 being up-regulated and 52 being down-regulated. Among the DEGs, several immune-related genes have been identified, which might play important roles in pathogen recognition and immune signaling.

C-type lectins (CTLs) are PRRs specifically recognize the sugar residues or motifs present in the glycans of pathogens (Mnich et al., 2020). In shrimp and other invertebrates’ immunity, CTLs play much more important roles than in mammals since the invertebrates lack adaptive immunity and largely depend on innate immunity to detect and combat pathogen invasion. Studies on shrimp C-type lectin also showed that they have multiple antibacterial activities, such as promoting phagocytosis, inhibiting bacterial attachment, regulating downstream immune effectors, and directing antimicrobial activity (Wang et al., 2020). In this research, among the up-regulated DEGs, two lectin-related genes, galactose-specific lectin nattectin-like (LOC113811918) and macrophage mannose receptor 1-like (LOC113819956), were found to increase their mRNA level in the △*flhG* infected shrimp hepatopancreas. In *Thalassophryne nattereri* fish, nattectin is involved in the Th1 responses and macrophage differentiation as a signal molecular (Lopes-Ferreira et al., 2011; Saraiva et al., 2011). The agglutinating activity against bacteria of nattectin was found in *Misgurnus anguillicaudatus* and *Carassius auratus* (Wang et al., 2017; Zhang et al., 2020). Lv et al. isolated a nattectin from *Larimichthys crocea* and investigated its role as a PRR in innate immunity (Lv et al., 2016). Another common C-type lectin, mannose receptor (CD206), also can bind to microbial surface glycan structures with terminal fucose, mannose, and N-Acetylglucosamine (GlcNAc) and active various immune cells (Van Der Zande et al., 2021). The mannose receptors of blunt snout bream (*Megalobrama amblycephala*) can recognize and mediate chitooligosaccharide internalization into macrophages (Ouyang et al., 2021). Xin et al. cloned and analyzed a mannose receptor from red swamp crayfish (Procambarus clarkii), investigating its role in bacteria binding and agglutination (Man et al., 2018). In our results, these two CTL genes’ up-regulated expression might relate to the presence of more flagella compared to the WT type group, suggesting that they have involved in bacteria flagella recognition and pathogen clearance.

The TNF receptor-associated factor (TRAF) 6-like in hepatopancreas also up-regulated significantly after being infected with the hyper flagella strain. TRAF proteins directly bind to the cytoplasmic tail of the tumor necrosis factor (TNF) receptor superfamily (TNFRSF) and there are seven members of the TRAF (TRAF1–7) (Ishida et al., 1996; Yamamoto et al., 2021). TRAF2, 5, and 6 activate nuclear factor-κB (NF-κB) and are involved in the canonical NF-κB pathway (Hayden and Ghosh, 2014). Multiple studies have elucidated TRAF6’s function as a molecular bridge that connects upstream TLRs, MyD88, and IRAKs to the downstream NF-kappa B and MAPK-signaling pathways (Kim and Rikihisa, 2002). In *Penaeus monodon*, *Pm*TRAF6 up-regulated constantly after being challenged by the white spot syndrome virus (WSSV) but remained unchanged after poly I:C stimulation (Deepika et al., 2014). Zhao et al. reported that MST4 which phosphorylates TRAF6 responded to *V. alginolyticus* infection and activated the TLR-TRAF6 signaling pathway to increase respiratory burst (RB) activity and decrease the total hemocyte count (THC) in *L. vannamei* (Zhao et al., 2017). However, according to the studies of Wang et al., the mRNA level of *Lv*TRAF6 in the *L. vannamei*’s hepatopancreas was at a lower level compared to other tissues and increased by the WSSV infection but unchanged after *V. alginolyticus* challenge (Wang et al., 2011). These findings imply that *V. alginolyticus* possesses a mechanism to evade the activation of the TRAF6 pathway. In this study, the transcriptome data show that the *flh*G mutant strains increased the gene expression of TRAF6 (LOC113810339) significantly than the WT strains of *V. alginolyticus* HN08155, suggesting that the TRAF6 signaling pathway is vital for the bacteria immune defense in shrimp and can be activated by the flagella.

## 5 Conclusion

The hepatopancreas of *L. vannamei* displayed distinct gene expression patterns after infection with a *V. alginolyticus* mutant strain that possessed constant hyper flagella, achieved through the deletion of the *flhG* gene, as compared to the wild type strain. Among the differentially expressed genes (DEGs), galactose-specific lectin nattectin and macrophage mannose receptor 1, both belonging to the CTL family, were found to potentially participate in flagella recognition. Additionally, the up-regulation of the TNF receptor-associated factor (TRAF) 6 gene in the hepatopancreas suggested the involvement of the TRAF6 pathway in immune activation.

## Data availability statement

The datasets presented in this study can be found in online repositories. The names of the repository/repositories and accession number(s) can be found in the article/**Supplementary Material**.

## Ethics statement

The manuscript presents research on animals that do not require ethical approval for their study.

## Author contributions

JZ: Writing – original draft. KL: Data curation, Writing – review & editing. XG: Writing – original draft. NZ: Writing – original draft. YZ: Writing – review & editing. WR: Writing – review & editing. AH: Writing – review & editing. HL: Supervision, Writing – review & editing. ZX: Supervision, Writing – review & editing.
