# Supplementary material for: Transcriptome analysis of the hepatopancreas from the Litopenaeus vannamei infected with different flagellum types of Vibrio alginolyticus strains
Source: Front Cell Infect Microbiol. 2023 Nov 21;13:1265917. doi: 10.3389/fcimb.2023.1265917 (PMC10703188; doi:10.3389/fcimb.2023.1265917)
Supplement: Supplementary file 1 [file Table_1.docx]

# Supplementary Data

### Table 1 Significantly DEGs of △*flhG* vs. WT

| Gene ID | Gene description | Fold change |
| --- | --- | --- |
| **up-regulated genes** |  |  |
| LOC113824731 | crustacyanin-C1 subunit-like | 462.189 |
| LOC113828299 | cyclin-dependent kinase inhibitor 1C-like | 8.865 |
| LOC113820947 | thymidylate kinase-like, transcript variant X1 | 4.521 |
| LOC113830401 | organic cation transporter protein-like | 48.408 |
| LOC113800085 | unknown | 157.428 |
| LOC113824722 | crustacyanin-A2 subunit-like | 329.041 |
| LOC113810339 | TNF receptor-associated factor 6-like | 75.605 |
| LOC113824721 | crustacyanin-C1 subunit-like | 21.056 |
| LOC113816916 | unknown | 197.936 |
| LOC113830402 | solute carrier family 22 members 7-like | 46.053 |
| LOC113816884 | beta-1,4-glucuronyltransferase 1-like | 44.519 |
| LOC113809120 | unknown | 2.353 |
| LOC113811681 | myc-associated zinc finger protein-like | 156.751 |
| LOC113802550 | triosephosphate isomerase B-like, transcript variant X1 | 24.558 |
| LOC113828559 | branchpoint-bridging protein-like, transcript variant X1 | 15.833 |
| LOC113824723 | crustacyanin-A2 subunit-like | 19.593 |
| LOC113824724 | crustacyanin-C1 subunit-like | 65.644 |
| LOC113818833 | excitatory amino acid transporter 1-like | 24.81 |
| LOC113814499 | probable nuclear hormone receptor HR3, transcript variant X1 | 45.414 |
| LOC113801169 | hormone receptor 4-like, transcript variant X1 | 18.122 |
| LOC113806961 | unknown | 115.227 |
| LOC113811918 | galactose-specific lectin nattectin-like | 12.148 |
| LOC113824737 | unknown | 52.941 |
| LOC113802205 | transcript variant X1 | 124.141 |
| LOC113827872 | transcript variant X1 | 19.454 |
| LOC113827839 | zyxin-like | 19.405 |
| LOC113801895 | unknown | 3.851 |
| LOC113806288 | leukocyte elastase inhibitor-like | 16.443 |
| LOC113815973 | protein unc-13 homolog 4B-like | 148.074 |
| LOC113828077 | unknown | 15.504 |
| LOC113819956 | macrophage mannose receptor 1-like, transcript variant X2 | 8.099 |
| LOC113802412 | unknown | 8.572 |
| LOC113828060 | unknown | 7.895 |
| LOC113816434 | UMP-CMP kinase-like, transcript variant X1 | 2.092 |
| LOC113800785 | endoribonuclease Dicer-like | 5.416 |
| LOC113820161 | chorion peroxidase-like | 78.343 |
| LOC113804797 | unknown | 33.417 |
| LOC113822187 | solute carrier family 15 member 2-like, transcript variant X1 | 8.885 |
| LOC113826005 | unknown | 272.045 |
| LOC113815331 | excitatory amino acid transporter 3-like, transcript variant X1 | 12.355 |
| LOC113819584 | 26S proteasome non-ATPase regulatory subunit 1-like | 2.621 |
| LOC113827756 | U3 small nucleolar ribonucleoprotein protein MPP10-like | 4.378 |
| LOC113824726 | crustacyanin-A2 subunit-like | 30.937 |
| LOC113824728 | crustacyanin-A1 subunit-like | 18.461 |
| LOC113808073 | transcript variant X1 | 6.008 |
| LOC113812066 | unknown | 3.093 |
| LOC113803329 | transcript variant X1 | 5.793 |
| LOC113827641 | unknown | 67.084 |
| LOC113807952 | unknown | 35.612 |
| LOC113806970 | unknown | 14.045 |
| LOC113804751 | sodium-dependent multivitamin transporter-like, transcript variant X2 | 64.212 |
| LOC113818498 | RNA-binding protein 28-like | 2.995 |
| **down-regulated genes** |  |  |
| LOC113811643 | probable cytochrome P450 301a1, mitochondrial | 0.026 |
| LOC113825357 | unknown | 0.096 |
| LOC113825012 | fatty acid binding protein 1-B.1-like | 0.002 |
| LOC113808505 | unknown | 0.378 |
| LOC113803966 | integumentary mucin C.1-like | 0.152 |
| LOC113810086 | unknown | 0.23 |
| LOC113807453 | beta, beta-carotene 15,15'-dioxygenase-like | 0.102 |
| LOC113826413 | unknown | 0.184 |
| LOC113800111 | peritrophin-1-like | 0.191 |
| LOC113823785 | trypsin-1-like | 0.032 |
| LOC113812532 | transient receptor potential cation channel trpm-like | 0.334 |
| LOC113823154 | cytochrome P450 4c3-like | 0.278 |
| LOC113822330 | unknown | 0.167 |
| LOC113804456 | thiopurine S-methyltransferase-like | 0.25 |
| LOC113817282 | carboxypeptidase B-like | 0.289 |
| LOC113816942 | endothelial lipase-like | 0.114 |
| LOC113811513 | trans-1,2-dihydro benzene-1,2-diol dehydrogenase-like, transcript variant X4 | 0.343 |
| LOC113800292 | alanine and glycine-rich protein-like | 0.277 |
| LOC113822335 | nitrate reductase [NADH] 1-like | 0.186 |
| LOC113825036 | unknown | 0.49 |
| LOC113817853 | putative inorganic phosphate cotransporter | 0.155 |
| LOC113827164 | glucose-6-phosphatase-like | 0.154 |
| LOC113804088 | unknown | 0.225 |
| LOC113815940 | fatty acid synthase-like | 0.388 |
| LOC113820472 | transcript variant X1 | 0.143 |
| LOC113824814 | pleckstrin homology domain-containing family G member 7-like | 0.443 |
| LOC113803689 | uncharacterized protein PF11_0207-like, transcript variant X1 | 0.076 |
| LOC113814341 | sorbitol dehydrogenase-like | 0.423 |
| LOC113817597 | unknown | 0.477 |
| LOC113816629 | unknown | 0.44 |
| LOC113820851 | group 3 secretory phospholipase A2-like | 0.241 |
| LOC113810566 | cytochrome P450 4C1-like, transcript variant X3 | 0.349 |
| LOC113814162 | unknown | 0.444 |
| LOC113813240 | facilitated trehalose transporter Tret1-like, transcript variant X1 | 0.147 |
| LOC113826143 | transient receptor potential channel pyrexia-like | 0.379 |
| LOC113812358 | sodium-independent sulfate anion transporter-like | 0.427 |
| LOC113801249 | acylcarnitine hydrolase-like | 0.413 |
| LOC113803018 | unknown | 0.262 |
| LOC113808752 | probable methylmalonate-semialdehyde dehydrogenase [acylating], mitochondrial | 0.463 |
| LOC113828152 | 4-aminobutyrate aminotransferase, mitochondrial-like | 0.305 |
| LOC113815619 | UDP-glucuronosyltransferase 2C1-like | 0.343 |
| LOC113823514 | 4-aminobutyrate aminotransferase, mitochondrial-like | 0.177 |
| LOC113829848 | glucose-6-phosphatase-like | 0.163 |
| LOC113810891 | unknown | 0.405 |
| LOC113810905 | holotricin-3-like, transcript variant X1 | 0.052 |
| LOC113807693 | cytochrome P450 2L1-like | 0.171 |
| LOC113807426 | disrupted in renal carcinoma protein 2 homolog, transcript variant X1 | 0.367 |
| LOC113817364 | proton-coupled folate transporter-like | 0.313 |
| LOC113809225 | chymotrypsin BI-like | 0.031 |
| LOC113817189 | cytosolic non-specific dipeptidase-like, transcript variant X1 | 0.294 |
| LOC113822449 | Unknown | 0.437 |
| LOC113812533 | repressed by EFG1 protein 1-like | 0.3 |
